# Standardization of a Protocol to Quantify Benzodiazepines in Human Urine Samples Employing Gas Chromatography–Mass Spectrometry—A Green Analytical Chemistry Approach

**DOI:** 10.1021/acsomega.6c06253

**Published:** 2026-07-28

**Authors:** Vibha Acharya, Shankar M Bakkannavar, Rajeev Jain

**Affiliations:** † Department of Forensic Medicine & Toxicology, Kasturba Medical College, 29224 Manipal Academy of Higher Education, Manipal 576104, India; ‡ Central Forensic Science Laboratory Directorate of Forensic Science Services, 563397 Ministry of Home Affairs, Government of India, Plot No. 2, Dakshin Marg, Sector 36A, Chandigarh 160036, India

## Abstract

Benzodiazepines (BZDs) are central nervous system depressants, prescribed to slow brain activity during anxiety, seizures, insomnia, etc. However, their long-term usage is associated with health risks such as physical and physiological dependence and cognitive decline. Poisoning due to BZDs is frequently encountered in forensic toxicology, which requires their rapid and robust detection. However, their low availability in biological samples and the lack of sensitive and rapid detection methods pose challenges. In this study, we standardized a protocol for the detection and quantification of lorazepam (LZ), clonazepam (CZ), and alprazolam (AZ), the short-to-intermediate and long-acting BZDs, without employing any derivatization steps and using gas chromatography–mass spectrometry (GC–MS). The detection limit (LOD) of BZDs by GC–MS was 5 ng/mL, and the detection was linear between 5 and 50 ng/mL, with coefficients of determination (*R* ^2^) of 0.9984, 0.9968, and 0.9936 for LZ, CZ, and AZ, respectively. Further, rapid dispersive liquid–liquid microextraction allowed enriching BZDs from human urine samples for following GC–MS analysis. Interday and intraday detection precisions were analyzed, which were within agreeable limits. Together, we have developed a green analytical chemistry protocol for the rapid detection of BZDs that can be employed in forensic toxicology and analytical chemistry laboratories for criminal cases, drug overdoses, or drug monitoring.

## Graphical abstract

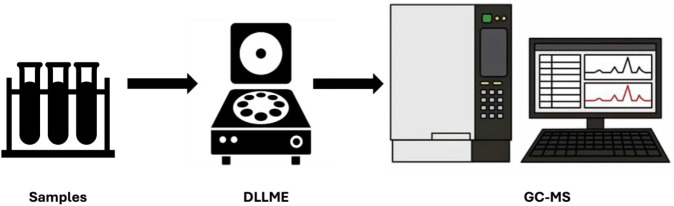

Benzodiazepines (BZDs) are extensively prescribed for a broad range of clinical indications, including use as adjuncts in general anesthesia and in the treatment of anxiety disorders, insomnia, and seizure disorders.^1^ The annual prevalence of BZD use varies widely, ranging from approximately 2%–17%, depending on the country and the methodological characteristics of individual studies.^2,3^ One of the major social issues that could arise globally is the overuse or abuse of BZDs. Given that long-term BZD use is associated with dependence, withdrawal phenomena, and adverse cognitive outcomes, including an increased risk of dementia, their continued prescription should be critically reevaluated beyond 12 weeks.^4–6^ Several studies have reported methods for the identification of BZDs and their metabolites in biological fluids.^7–9^ These investigations employ chromatographic or immunological approaches.^10–25^ Liquid chromatography (LC) is the most used technique for BZD analysis.^7,10,11,20,23,24^ While some of these methods require sophisticated and costly LC instrumentation in conjunction with single^18–20^ or tandem mass spectrometry (TMS),^7,10,11,14,21,22^ others use UV/vis^15,22,25^ or diode-array detectors^15^ and have lower specificity.

Urine is one of the preferred biological specimens for the determination of drugs and pharmaceuticals owing to its ease of collection and the relatively high concentrations of analytes present. As drugs are typically excreted in urine after metabolic conversion, the analysis of metabolites plays a central role in urine-based testing. Many of these metabolites are still active and have therapeutic benefits when it comes to BZDs.^26^

Prior to instrumental analysis, sample preparation is crucial for the detection of trace-level analytes in complex matrices.^27^ The principles of Green Analytical Chemistry (GAC) are increasingly emphasized in contemporary sample preparation methodologies. The primary goal of GAC is to develop new analytical techniques that will reduce the use of reagents (perhaps by using low-toxicity and biodegradable solvents), minimize waste, use the least amount of energy, and ensure the safety of the operator and analyst.^28^ Analysts have shown a great deal of interest in Dispersive Liquid–Liquid Microextraction (DLLME) because of its rapidity, high enrichment factors, cost, convenience of use, and environmental friendliness.^29,30^ DLLME is carried out following the injection of the disperser and extraction solvents into an aqueous phase. The process yields a turbid solution characterized by fine droplets of extraction solvent dispersed within the aqueous phase. Following centrifugation, the dense extraction solvent settles as a sedimented phase and is subsequently subjected to further analysis.^31^ The increasing number of applications in domains like forensic, clinical, environmental, and pharmaceutical analysis, among many others, points to the expanding popularity of DLLME.^32–37^

In the present study, a gas chromatography–mass spectrometry (GC–MS)-based, sensitive, and reliable approach was developed and validated in this study to determine three BZDs: lorazepam (LZ), clonazepam (CZ), and alprazolam (AZ). The use of Dispersive Liquid–Liquid Microextraction (DLLME) as a quick, affordable, and eco-friendly sample preparation method was given particular focus. Compared to traditional extraction methods like liquid–liquid extraction or solid-phase extraction, the DLLME greatly reduced solvent consumption, sample handling, extraction time, and analytical waste generation by requiring only a small volume of urine sample and a few microliters of extraction and disperser solvents.

To increase the volatility of the analyte, thermal stability, or chromatographic behavior, many previously developed GC–MS methods involved derivatization with certain reagents. However, the major advantage of the method developed here is that it does not require any derivatization before the analysis. The eliminated derivatization step was found to be advantageous in many ways, such as decreased analysis time, minimal reagent consumption, avoidance of potential errors, and minimal sample preparation.

This demonstrates how the suggested approach can be used for routine forensic and toxicological analysis of BZDs in a simple, rapid, economical, greener, and environmentally friendly process.

## Materials

### Chemicals and Reagents

Reference standards of drugs were procured from the Indian Pharmacopoeia Commission (IPC), Ghaziabad, and stored at room temperature. Analytical grades of chloroform (CF), acetone (ACE), and methanol (MeOH) were procured from Sigma-Aldrich. Anonymized human urine samples suspected of BZD overdose were collected from a toxicology laboratory and analyzed.

## Methods

### Instrumentation

Shimadzu GC–MS QP-2020 NX GC–MS instrument was used for the analysis. The injector temperature was set to 250 °C. The injection mode was splitless. The sampling time was 1.00 min. The column flow pressure was set to 131.7 kPa. The total flow and column flow were maintained at 40.2 mL/min and 1.20 mL/min, respectively. ***Column parameters***: The initial temperature was set at 90 °C, held for 4 min, and then raised to 300 °C at a rate of 20 °C/min and held for 10 min. The total run time was 24.50 min. ***MS Parameters***: The ion source temperature and interface temperature were both set at 250 °C. The solvent cut time was 2 min. The acquisition mode was SIM.

### Stock Solutions and Working Solutions

Prior to the analysis, a 1000 ng/mL stock solution of all three drugs, LZ, CZ, and AZ, was prepared in methanol and stored at −4 °C. Serial dilutions were made to prepare working solutions ranging from 1 to 100 ng/mL in methanol for calibration.

### Sample Extraction Procedure

Institutional Ethics Committee approval (IEC no. 326/2022) was obtained prior to the collection of anonymized urine samples from the toxicology laboratory.

The best extraction efficiency for DLLME was determined for the four most often used disperser solvents—methanol (MeOH), ethanol (EtOH), acetone (ACE), and acetonitrile (ACN)—and the four most often used extraction solvents: dichloromethane (DCM), carbon tetrachloride (CCl4), chloroform (CF), and chlorobenzene (CB). The volume of the disperser and extraction liquids was optimized in future trials. The disperser solvent optimized in this study was measured in various volumes, including 200, 400, 600, 800, and 1000 μL. The extraction solvent used in the current study, was administered in various concentrations, including 100, 200, 300, and 400 μL. To determine the influence of ultrasonication time on DLLME extraction efficacy, a series of studies were performed with ultrasonication periods ranging from 30 to 180 s (30, 60, 90, 120, 150, and 180 s). A series of trials were conducted to optimize the final pH (4, 6, 8, 10, 12) and ionic strength (0%, 5%, 10%) of the sample to attain maximum extraction. Several trials were conducted to optimize vortex time and speed, which ranged from 30 to 120 s (30, 50, 70, 90, 120 s) and 100 to 1000 rpm (100, 300, 500, 700, 900, 1000 rpm), respectively.

Pure standard drug solution or urine sample (1 mL) was diluted in 5 mL of distilled water. DLLME was performed by plunging the mixture of disperser solvent and extraction solvent using a syringe into the mixture. The mixture was then centrifuged. The supernatant was discarded, and 100 μL of the sediment, in the form of a droplet, was added to 900 μL of methanol and used for GC–MS analysis.

### Optimization of GC–MS Conditions

Optimized conditions were used to validate the developed method for the qualitative and quantitative evaluation of all three BZDs. A pure standard of all three BZDs was run and scanned to determine the runtime. LZ, CZ, and AZ standards were processed as samples by spiking the urine matrix, and calibration curves were developed.

### Bioanalytical Method Validation

The method for qualitatively and quantitatively determining LZ, CZ, and AZ from urine was optimized and validated for its linearity, specificity, limit of detection (LOD), limit of quantification (LOQ), accuracy, precision, recovery, stability, and carryover.

***Linearity and sensitivity***: The standard solutions of the three drugs, which ranged from 1 ng/mL to 100 ng/mL, were used to assess the linearity of the developed approach. After the injection of triplicates of each standard, the linearity range, the limit of detection (LOD), the limit of quantitation (LOQ), and the RSD (%) were determined.
***Selectivity and Specificity:*** The calibration standards and urine-spiked matrix samples were extracted in accordance with the usual procedure.
***Accuracy and precision:*** The established method for the qualitative and quantitative determination of LZ, CZ, and AZ was evaluated for accuracy and precision by analyzing standards in triplicate. Accuracy was assessed on both intraday and interday bases using calibration curve standards and spiked urine samples.
***Recovery***: By comparing the peak area of the drugs in the extracted urine sample at LQC, MQC, and HQC to the peak area of the blank, which was spiked with the three drugs after extraction, the recovery of the developed method was assessed in accordance with conventional guidelines.
***Carry over:*** Carry-over was assessed by analyzing a blank sample after the highest calibrator/spiked matrix for 5 days.
***Stability studies:*** The stability of all three drug stock solutions was determined based on the effect of storage at room temperature for a week. Freshly prepared stock solutions were used to compare the stability of the stored drug solutions. Percentage recovery was used to represent the stability of the stored stock solutions.

## Results

### Sample Extraction

Of allthe disperser solvents that were studied, acetone had the best extraction efficiency. As a result, acetone was chosen as the disperser solvent for further experiments. Chloroform outperformed all of the other extraction solvents tested. Chloroform was used as the extraction solvent for all subsequent trials. The maximal extraction of BZDs by DLLME was achieved using 600 μL of acetone. Therefore, 600 μL of acetone was used as the disperser solvent. BZDs were extracted very effectively using 200 μL of chloroform. As a result, the subsequent experiments used 200 μL of chloroform as the extraction solvent (Figure 1).

**Figure 1:**
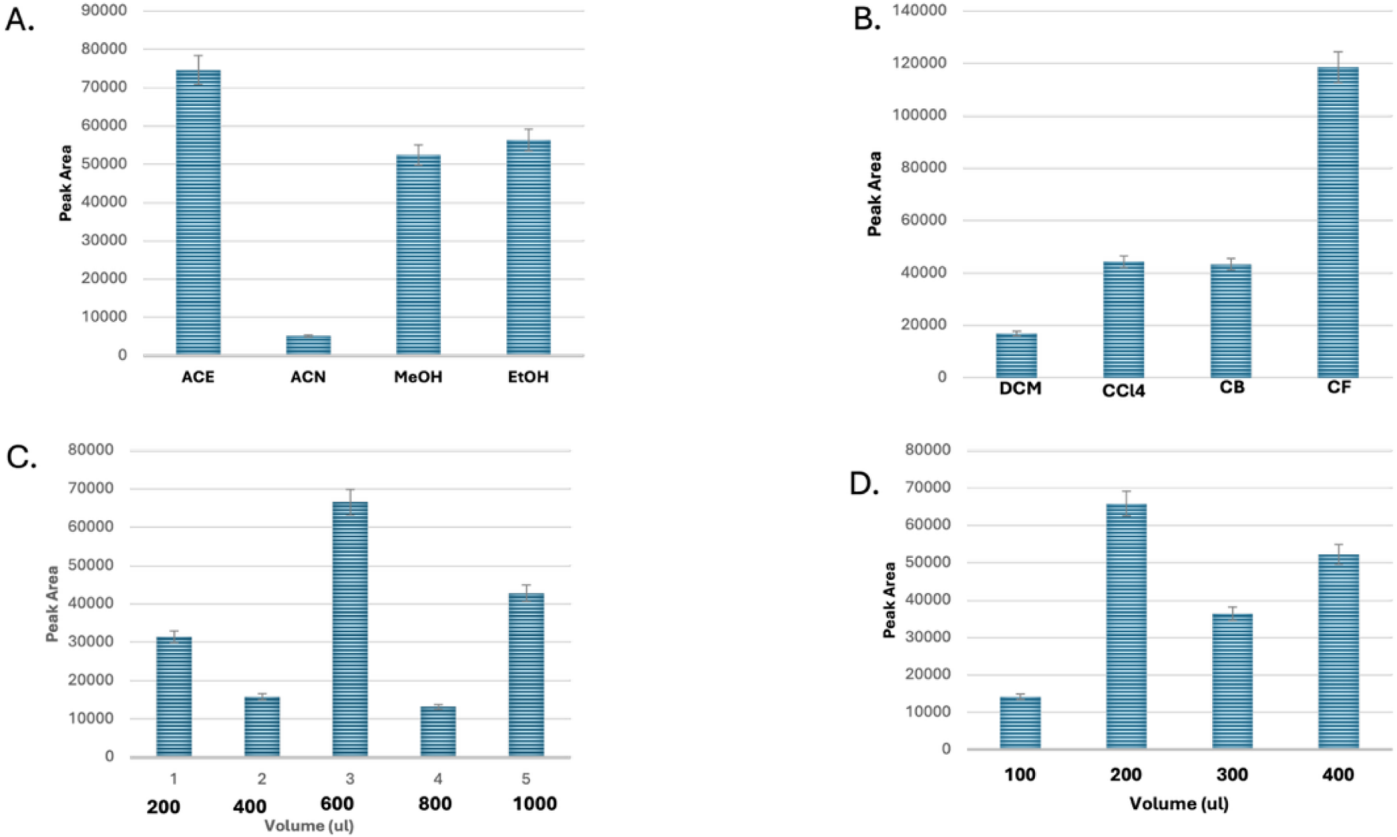
A. Acetone optimized as the disperser solvent. B. Chloroform optimized as the extraction solvent. C. The volume of the disperser solvent, i.e., 600 μL of acetone. D. The volume of the extraction solvent, i.e., 200 μL of chloroform.

pH was optimized at 12, and the ionic strength was maintained at 0%. The maximum DLLME extraction efficacy was achieved with 60 s of ultrasonication. The best extraction of all three BZDs was obtained after 70 s of vortex mixing at 500 rpm.

Standard/sample (1 mL) was diluted with 5 mL of distilled water and subjected to the DLLME procedure, plunging a mixture of 600 μL of acetone and 200 μL of chloroform using a syringe. The mixture was then centrifuged at 3000 rpm for 10 min. The supernatant was discarded, and 100 μL of the settled droplet was transferred to a fresh vial, diluted with 900 μL of methanol, and used for GC–MS analysis in SIM mode with the above-mentioned GC and MS parameters. Excellent separation was obtained between the three target BZDs, with optimal peak characteristics (Figure 2). Subsequently, the bioanalytical method validation was carried out using the SIM mode.

**Figure 2:**
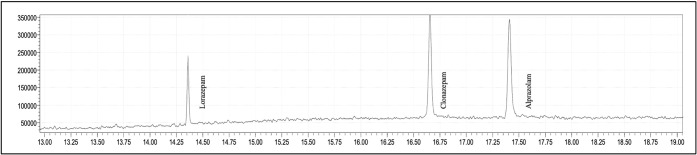
Chromatogram shows the separation of target BZDs from a mixture following the proposed DLLME procedure.

### Bioanalytical Method Validation

(a) ***Linearity and sensitivity:*** Calibration curves were obtained for all three drugs in a single run in the urine matrix, and they were found to be linear in the range of 5–50 ng/mL. The curves for LZ, CZ, and AZ were plotted with peak area on the *y*-axis and concentration on the *x*-axis. The values of slope, intercept, and *R* ^2^ were obtained using a linear regression method. The *R* ^2^ values were 0.9984, 0.9968, and 0.9936 for LZ, CZ, and AZ, respectively. ***(b) Selectivity and Specificity:*** No peak was found for either blank or blank matrix samples, indicating that the technique is specific and selective for LZ, CZ, and AZ, with no interference from biological matrix components or extraction agents. ***(c) Accuracy and Precision:*** The intraday and interday accuracy and precision of the developed method for the target drugs are represented in Table 1. Low-quality control (LQC), mid-quality control (MQC), and high-quality control (HQC) were determined to be within acceptable limits, with all levels ranging between 1% and 15%. ***(d) Recovery:*** The mean recovery of the target BZs, i.e., LZ, CZ, and AZ, in the urine matrix at the designated calibrator concentrations was found to be in the range of 61%–118% (Table 1). ***(e) Carry over:*** Carry over was assessed by analyzing blank samples after the highest calibrator/spiked matrix for 5 days and was found to be well within the acceptable guidelines (peak area not exceeding 20% of LOQ). ***(f) Stability studies:*** The standards were stored at room temperature for 7 days and showed a recovery of 111.448% at 10 ng/mL, 95.879% at 50 ng/mL, and 100.961% at 100 ng/mL for LZ; 120.456% at 10 ng/mL, 92.637% at 50 ng/mL, and 101.637% at 100 ng/mL for CZ; and 110.032% at 10 ng/mL, 96.388% at 50 ng/mL, and 100.802% at 100 ng/mL for AZ (Table 2).

**Table 1:** Linearity Range, LOD, LOQ, Accuracy, Precision, Room Temperature Stability of BZDs in Urine Matrix

				Accuracy (%Bias)^a^			Precision (%RSD)^a^			Recovery %^a^		
Drug	Linearity range (ng/mL)	LOD (ng/mL)	LOQ (ng/mL)	LQC	MQC	HQC	LQC	MQC	HQC	LQC	MQC	HQC
**LZ**	5–50	1.8 ng/mL	5 ng/mL	0.008	2.56	0.03	0.24	5.90	0.27	99.91	99.99	61.86
**CZ**	5–50	1.5 ng/mL	5 ng/mL	–0.04	0.12	–0.02	10.4	7.86	8.90	104.65	87.61	102.41
**AZ**	5–50	1.4 ng/mL	5 ng/mL	–0.09	–0.14	0.04	0.76	3.01	1.98	109.61	118.43	95.81

a LQC= low-quality control (7.5 ng/mL), MQC = mid-quality control (25 ng/mL), HQC = high-quality control (50 ng/mL).

**Table 2:** Room Temperature Stability of BZDs in the Urine Matrix

Room temperature stability (Recovery %)^a^		
LQC	MQC	HQC
111.44	95.87	100.96
120.45	92.63	101.63
110.03	96.38	100.80

a LQC= low-quality control (10 ng/mL), MQC = mid-quality control (50 ng/mL), HQC = high-quality control (100 ng/mL).

### Sample Analysis

Urine samples (12), tested positive for LZ, CZ, and AZ, were collected from the toxicology laboratory and analyzed using GC–MS. Sample preparation was done using DLLME, which efficiently extracted and concentrated BZDs and was run under GC–MS without derivatization. The measured concentration of LZ in the samples ranged from 2.34 to 7.92 ng/mL, CZ in the samples ranged from 4.85 to 7.12 ng/mL, and AZ in the range of 1.05–6.86 ng/mL (Table 3).

**Table 3:** Results of Quantification of Urine Samples Tested Positive for LZ, CZ, and AZ

Sample No:	Lorazepam (ng/mL)	Clonazepam (ng/mL)	Alprazolam (ng/mL)
01.	6.34	5.37	1.66
02.	7.02	6.63	2.94
03.	5.82	4.85	4.42
04.	5.66	7.07	6.86
05.	6.63	5.59	3.02
06.	5.24	6.05	1.05
07.	4.68	6.90	2.53
08.	5.14	5.22	1.66
09.	3.36	4.08	2.63
10.	7.92	6.53	3.24
11.	2.34	7.12	4.55
12.	2.98	6.63	1.64

## Discussion

In this study, an eco-friendly, robust, and cost-effective derivatization-free method is developed and validated for the quantification of three BZDs (LZ, CZ, and AZ) in human urine using DLLME coupled with GC–MS. The primary focus of clinical and forensic toxicology laboratories is the identification of drugs of abuse related to various chemical classes (opiates, cocaine, amphetamines, hallucinogens), BZDs, and antipsychotic drugs that may be involved in intoxications (which are frequently fatal), drug-facilitated crimes, or traffic controls (driving under the influence of drugs) in biological samples. Forensic toxicology, for instance, analyzes a variety of extremely complicated biological specimens, including blood, plasma, urine, oral fluid, and hair extract. The inherent complexity of the matrix and the generally low concentration of BZDs in forensic biological samples make it difficult to identify; therefore, sensitive and quick procedures must be developed.^38,39^

Due to the noninvasive sampling technique, low requirement for experts, and large sample output, urine analysis has become a valuable tool for assessing and tracking drug intake. Urine is especially helpful for toxicological examinations because of its broad detection window, which can extend from minutes to days or even weeks. However, drugs are typically found in urine at trace amounts (μg/mL or ng/mL), requiring extremely sensitive testing techniques.^38^ Sample preparation and instrumental analysis must be carefully developed and used to concentrate the sample and obtain precise and sensitive detection in order to overcome these difficulties. The development of pretreatment techniques for BZD analysis in biological samples currently depends on a number of cutting-edge subfields of liquid–liquid microextraction (LLME) and solid-phase microextraction (SPME), which are selected due to their inherent benefits (such as execution time, complexity, and cost) and matrix type compatibility.^40^

DLLME is a quick and inexpensive technique that can efficiently purify specimens for the simultaneous extraction of several drugs from different classes. As an alternative to conventional LLE, DLLME has become a popular green pretreatment method for drug extraction because it uses smaller volumes of organic solvents (μL range).^39,41–43^ This approach was first used in environmental analysis, food analysis,^44–48^ and biological samples for the determination of prescription medications^49,50^ and forensic toxicology for the determination of drugs of abuse in human plasma,^51^ blood,^52^ and urine.^53–55^ These latter studies, which optimized the extraction conditions and pioneered the use of DLLME in forensic fields, were limited to a single class of analytes, such as six drugs of abuse^52^ or seven BZDs in plasma,^49^ opium alkaloids^53^ and amphetamines in urine,^54^ or a single compound like amitriptyline phosphatidylethanol^50^ and fentanyl.^56^ The simultaneous DLLME of roughly 30 substances of abuse from a urine test is covered in only one study.^57^

Choosing the right extraction solvent is critical in DLLME because it has a direct impact on the preconcentration factor and the extraction yield. The ideal extraction solvent should be immiscible with water, have a density higher than that of water, and be capable of extracting the desired analytes. It should also exhibit sufficient chromatographic characteristics when it is detected on a TLC plate. Four different extraction solvents with densities higher than that of water were evaluated to identify the most suitable extraction solvent. In accordance with the principles of conventional DLLME, chloroform was included owing to its well-established extraction performance and extensive application in the extraction of benzodiazepines (BZDs). Although alternative green solvents, such as deep eutectic solvents (DESs) and ionic liquids, have gained attention due to their environmentally favorable characteristics, their practical application remains limited by challenges associated with synthesis complexity, preparation procedures, and relatively high commercial costs. Therefore, conventional solvents remain advantageous in terms of their accessibility, ease of use, and cost-effectiveness. Hence, chloroform was optimized as the final extraction solvent.

pH is one of the most critical variables that affect DLLME. The best reaction occurs at pH 6 and 8. This was expected, given that BZDs are weak bases. Thus, we were able to extract BZDs in the pH range of 4 to 12 by combining sodium hydroxide and hydrochloric acid in an aqueous solution. This allowed us to investigate the effect of the aqueous-phase pH. The best DLLME extraction efficiency was found when the aqueous-phase pH was set to 12, indicating that it was a weak base. The ionic strength was also examined by adding NaCl to the aqueous phase at concentrations ranging from 0% to 10%. When no salt was added, the extraction efficiency improved. Ultrasonication enhances the emulsification of the extraction solvent in the aqueous phase, as well as the subsequent mass transfer of the analyte from the aqueous phase to the extraction solvent. As the sonication time increases, the local temperature of the solution rises due to several cycles of compression and rarefaction. The extraction solvent is distributed into the aqueous samples via vortex mixing, which is a basic emulsification process.

Traditional analysis of GC–MS for BZDs includes derivatization.^58–61^ The thermal stability of most BZDs, which deteriorate quickly in the absence of previous derivatization, is the primary cause of several issues with the use of GC. However, emergency toxicological analysis is poorly adapted for this stage, which is frequently lengthy and delicate.^1^ On the contrary, as there was no derivatization involved in the current study, analytes other than BZDs were not detected in GC–MS. Therefore, the DLLME-GC–MS method optimized and validated here is very specific for BZDs. The advantages range from reduced sample preparation time, improved method reproducibility, to a lower risk of improper or incomplete sample preparation. The developed method is a significant advancement in the field of BZD analysis using GC–MS without derivatization.

### Evaluation of Matrix Interferences in the DLLME Procedure

The extracts obtained from urine samples were also tested for total protein and lipid content, which approximately measured up to 0.48 and 0.076 mg/mL, respectively. Although they were extracted along with BZDs during DLLME, they were, however, not detected in GC–MS (Table 4).

**Table 4:** A Comparative Table Including Parameters Such as Extraction Solvent, Sample Preparation, Linear Range, LOQ, and Recovery of Previous Studies for Quantification of BZDs

Reference	Extraction method/solvent	Sample preparation	Linear range	LOQ	Recovery (%)
^61^	LPME	Hydrolysis using sodium acetate and β-glucuronidase	50–250 ng/mL	0.5 to 30 ng/mL	3.30 to 92.77%
^62^	LLE Chloroform–isopropanol (9:1)	β-glucuronidase hydrolysis + derivatization	50–1000 ng/mL	10–50 ng/mL	70–90%
^63^	Organic solvent LLE (sodium bicarbonate + methylene chloride)	Hydrolysis	10–500 ng/mL	5–20 ng/mL	70–85%
^64^	SPE	Protein precipitation	250–750 ppb	250 ppb	80–90%
^58^	LLE with chloroform	Derivatization using (propylation and (propionylation)	25 ng/mL- 200 ng/mL	1.58–177.2 ng/mL	>74%
^65^	LLE with ethyl acetate	No derivatization	1–250 ng/mL	1–10 ng/mL	83.8% – 111.2%
^66^	SPE	Derivatization ethyl acetate and MTBSTFA	20–1000 ng/mL	6.13 ng/mL	44–83%
^67^	LLE isobutyl alcohol: methylene chloride solution (1:9)	β-glucuronidase hydrolysis	250–2000 ng/mL	170 ng/mL	comparable to that of the standards.
^68^	LLE	Enzymatic Hydrolysis	50–1500 ng/mL/3000 ng/mL	25–50 ng/mL	70–90%
^69^	LLE	Silylation	1–8000 ng/mL	1 to 100 ng/mL	3–100.8%
^70^	LLE	Enzymatic hydrolysis	1 ng/mL to 500 ng/mL	1 ng/mL	75% to 95%

### Assessment of the Practicality of the Proposed Method

A new GAC metric called the BAGI (Blue Applicability Grade Index) was introduced by Manousi et al. to assess the viability and environmental friendliness of analytical techniques. BAGI indicates the greenness of the tested assays using a pictogram, a color scale, and a score. This GAC metric has a score range from 25 to 100. Better assay greenness is indicated by a higher BAGI metric score. BAGI makes it simple and quick to assess the greenness of analytical assays^71,72^ (Figure 3).

**Figure 3:**
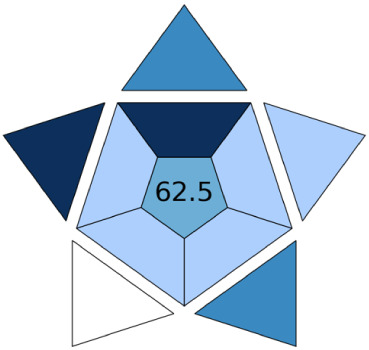
The practicality of the method is depicted by BAGI.

With a balanced performance in terms of analytical capability, operational feasibility, and laboratory implementation, the developed technique received a BAGI score of 62.5, indicating a satisfactory practical application. This demonstrates that the suggested method has good analytical utility and practical applicability, with strengths in sensitivity, multianalyte capability, and laboratory feasibility; however, its overall practicality is moderately impacted using sophisticated equipment and preconcentration requirements.

### Assessment of the Greenness of the Method

The AGSA (Analytical Green Star Area) metric offers a thorough and organized assessment of the greenness of analytical techniques through methodical scoring, and it is in line with the 12 Principles of GAC. The scoring system is intended to assess certain facets of the analytical process. Specific questions with three-tiered answer possibilities are used to evaluate each principle, enabling a gradual separation between approaches. Greater sustainability is indicated by higher ratings, which favor waste minimization techniques, nonhazardous chemicals, minimal sample treatment, and low energy use^73^ (Figure 4).

**Figure 4:**
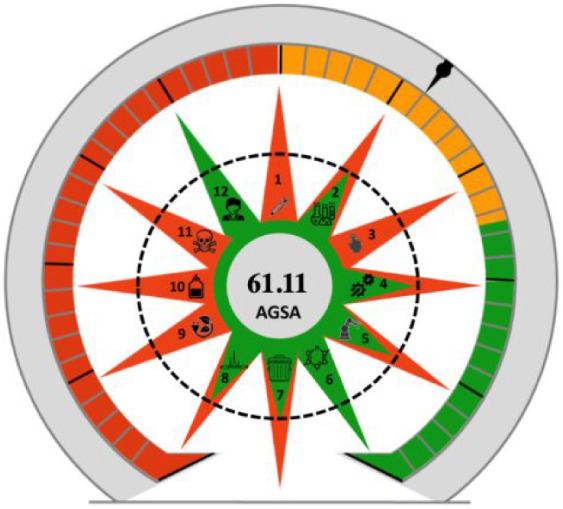
The greenness of the method is depicted by AGSA.

The method developed achieved an AGSA of 61.11, which indicated moderate to good agreement with green analytical chemistry principles. The score shows a balance between the analytical efficiency of the method and environmental sustainability. The greenness score is partly lowered by the stringent sample treatment requirements, which include extraction before analysis. However, by lowering the need for solvents, sample handling, and waste production, the use of a fully miniaturized extraction technique helps make up for this restriction. Effective sample usage is supported by the comparatively small necessary sample amount (0.1–1 g or similar sample volume). Instead, analysis is carried out in a remote laboratory environment, which reduces mobility and real-time monitoring capabilities. However, the approach shows a partial integration of analytical processes, which improves workflow efficiency by lowering the number of procedural stages and limiting instrument utilization. The absence of any derivatization for the developed method saves costs on additional reagents and lengthy modifications of chemical reactions, which may be considered a practical advantage. Moreover, less waste is generated during each analysis (<100 mL/sample), and adequate waste management policies are followed. High energy consumption during the analysis process (>1.5 kW/sample) is primarily associated with the utilization of GC–MS and may negatively affect the environmental performance of the analyzed procedure. As in this case, the AGSA score drops when the analysis involves nonrenewable reagents or substances belonging to hazard categories (≥6 pictograms). The method creates an operating environment with minimal risks, where there is no need for a substantial amount of personal protective equipment (PPE), thus ensuring operator safety. The total AGSA value of 61.11 suggests that the proposed technique is environmentally friendly and applicable to a considerable extent. While the sustainability of the technique is limited by some aspects, such as sample pretreatment, consumption of energy, and the use of nonrenewable and poisonous reagents. At the same time, it also benefits from miniaturization, elimination of derivatization, reduced waste generation, and multianalyte capability (Table 5(.

**Table 5:** Comparative Table with Various Studies for BZD Estimation with Solvent-Based Microextraction and Their Greenness Score

Reference	Method	Matrix	Accuracy	Precision	LOQ (ng/mL)	Greenness Score
^74^	LLME-HPLC-UV	Plasma & Urine	81–92%	<10%	2.3–11.2	87.8
 ^75^	PALME-UHPLC-MS/MS	Whole blood	52–104%	<15%	3.2–160	91.3
 ^76^	PALME-UHPLC-MS/MS	Whole blood	10–138%	<20%	1–5	88.6
 ^77^	EME-UHPLC-MS/MS	Plasma	38–74%	NA	5	87.6
 ^78^	DES-USAEME-UV	Saliva	87–113%	<10%	0.06–0.15	95.1

All three BZDs could be reliably identified and separated. In every test run, the retention time remained constant. Since there was no matrix interference of any kind, the approach is sensitive and specific for the BZDs. The turnaround time is another benefit of the developed method. For LZ, CZ, and AZ, the retention times are 14.35, 16.65, and 17.40 min, respectively. The extraction process took about 15 min. In contrast to previously reported methods, analysis of urine samples positive for these BZDs was completed within 1 h.^58,79,80^ Despite being a complicated matrix, the approach demonstrated outstanding linearity, precision, and accuracy. The approach completely aligns with the principles of the GAC. DLLME uses less solvent and produces less waste because it is a microscale extraction method. The method eliminates the use of toxic derivatization reagents by avoiding derivatization altogether. In general, less time and energy are used.

Compared with traditional GC–MS procedures, the approach is safer and more ecologically friendly. This technique can be used to monitor dependency on drugs and for routine clinical and forensic toxicological analysis. The analysis of other BZDs and testing in different biological matrices, such as plasma and blood, are potential future directions for this work.

The proposed method achieved LOD and LOQ comparable to those reported in existing GC–MS-based BZD assays. Although LCMS/MS-based methods may offer lower detection limits, GC–MS remains a robust and widely accessible platform, particularly in laboratories where derivatization-free or cost-effective analytical workflows are preferred. The DLLME approach employed in this study provides efficient analyte enrichment with minimal solvent consumption and short extraction times. This technique offers a practical advantage over conventional LLE- and SPE-based extraction methods. The absence of significant carryover further supports the suitability of the method for batch analysis and routine laboratory workflows. Such over-recoveries may be attributed to matrix-induced signal enhancement, solvent evaporation effects, or analyte partitioning behavior during the DLLME process. Importantly, the reproducibility of these recoveries across concentration levels supports the reliability of the extraction and analytical procedure.

The proposed method has certain limitations despite the robustness demonstrated. These include the absence of isotopically labeled internal standards, which may limit correction for matrix effects and extraction variability. An internal standard was intentionally not incorporated into the analytical protocol to maintain procedural simplicity and minimize sample preparation steps, thereby enhancing the practicality and adaptability of the method. Quantification using an external standard approach demonstrated satisfactory repeatability, reproducibility, precision, and accuracy, with all validation parameters remaining within acceptable limits, indicating minimal analytical variability. Ideally, stable isotope-labeled analogues of the target analytes are preferred as internal standards due to their ability to compensate for matrix effects and analytical fluctuations. However, their application in the present study was limited by financial constraints and restricted accessibility. Future studies can explore long-term storage stability, freeze–thaw stability, and autosampler stability of the analytes, and the method can be expanded to include BZD metabolites, such as glucuronide conjugates or hydroxylated derivatives, which could enhance its forensic and clinical utility, particularly for the interpretation of exposure timing and metabolic patterns.

Since there was no enzymatic or chemical step employed in order to conduct hydrolysis, the analytical approach utilized in the current study was aimed at quantifying the free, unconjugated forms of the BZDs in urine. The alkaline or acid hydrolysis step was omitted to render the method simpler, safer, and greener. The high degree of excretion of various BZDs and their metabolites in glucuronide conjugates is rather well-documented. As a result, the presence of such an unhydrolyzed form does not allow detection of these conjugates by using the current approach, and thus, the actual concentration of the substance corresponds solely to the free fraction.

On the whole, the proposed validated DLLME-GC–MS method provides a reliable, selective, and reproducible approach for the determination of three commonly prescribed BZDs in urine, meeting key regulatory performance criteria as per FDA and EMA guidelines. Its operational/analytical simplicity, attributed to the derivatization-free extraction workflow and efficient extraction recovery, makes it favorable for laboratories operating under resource-constrained settings while still adhering to internationally accepted bioanalytical standards. While the inclusion of isotopically labeled internal standards and long-term stability assessments could further strengthen quantitative robustness, the current method represents a pragmatic and analytically practical approach for BZD quantification.

## Conclusion

Overall, the GC–MS approach for BZD quantification presented here is straightforward, effective, and environmentally friendly. For routine BZD analysis without derivatization, this is a scalable and useful method.
